# Selective delignification of poplar wood with a newly isolated white-rot basidiomycete *Peniophora incarnata* T-7 by submerged fermentation to enhance saccharification

**DOI:** 10.1186/s13068-021-01986-y

**Published:** 2021-06-12

**Authors:** Jiangshan Ma, Huimin Yue, Hongqian Li, Jing Zhang, Yanghong Zhang, Xiaoling Wang, Si Gong, Gao-Qiang Liu

**Affiliations:** 1 Hunan Provincial Key Laboratory of Forestry Biotechnology, Central South University of Forestry and Technology, Changsha, 410004 Hunan P.R. China; 2 International Cooperation Base of Science and Technology Innovation on Forest Resource Biotechnology, Central South University of Forestry and Technology, Changsha, 410004 Hunan P.R. China

**Keywords:** Woody biomass, Fungal pretreatment, White-rot basidiomycete, *Peniophora incarnate* T-7, Submerged fermentation, Enzymatic saccharification, Quantitative proteomic analysis

## Abstract

**Background:**

Pretreatment is a critical step required for efficient conversion of woody biomass into biofuels and platform chemicals. Fungal pretreatment is regarded as one of the most promising technology for woody biomass conversion but remains challenging for industrial application. The exploration of potential fungus strain with high efficient delignification and less processing time for woody biomass pretreatment will be valuable for development of biorefinery industry. Here, a newly isolated white-rot basidiomycete *Peniophora incarnate* T-7 was employed for poplar wood pretreatment.

**Results:**

The chemical component analysis showed that cellulose, hemicellulose and lignin from poplar wood declined by 16%, 48% and 70%, respectively, after 7 days submerged fermentation by *P*. *incarnate* T-7. Enzymatic saccharification analysis revealed that the maximum yields of glucose and xylose from 7 days of *P*. *incarnate* T-7 treated poplar wood reached 33.4% and 27.6%, respectively, both of which were enhanced by sevenfold relative to the untreated group. Fourier transform infrared spectroscopy (FTIR), scanning electron microscope (SEM), X-ray diffraction (XRD) and pyrolysis gas chromatography–mass spectrometry (Py-GC/MS) characterization confirmed that lignocellulosic structure of poplar wood was largely broken by *P*. *incarnate* T-7, including delignification and de-crystalline of cellulose. Meanwhile, lignin component of poplar wood was selectively degraded by *P*. *incarnate* T-7, and G-type unit of lignin was preferentially attacked by the strain. Furthermore, quantitative proteomic analysis revealed that a considerable amount of lignocellulolytic enzymes were detected in the secretory proteins of *P*. *incarnate* T-7, especially with high abundance of lignin-degrading enzymes and hemicellulases. Combination of quantitative proteomic with transcriptomic analysis results showed that most of those lignocellulolytic enzymes were highly upregulated on poplar wood substrate compared to glucose substrate.

**Conclusions:**

This study showed that *P*. *incarnate* T-7 could selectively delignify poplar wood by submerged fermentation with short time of 7 days, which greatly improved its enzymatic saccharification efficiency. Our results suggested that *P*. *incarnate* T-7 might be a promising candidate for industrial woody biomass pretreatment.

**Supplementary Information:**

The online version contains supplementary material available at 10.1186/s13068-021-01986-y.

## Background

As the most abundant renewable terrestrial carbon source, lignocellulosic biomass primarily consisting of polysaccharides (cellulose and hemicellulose) and lignin, has been regarded as a promising resource for production of biofuels and platform chemicals that could partially substitute fossil carbon resources [1, 2]. Cellulose and hemicellulose components of biomass could be converted to fermentable hexose and pentose, respectively, by corresponding enzymes or chemical methods [3, 4]. While lignin is an amorphous and complex hetero-polymer consisting of three different phenyl-propane units with various ether and C–C bonds, which forms a matrix by embedded the spaces between cellulose and hemicellulose structures. These enabled lignin to function as a recalcitrance physical barrier of polysaccharides against biological and physical attacks [5–7]. Woody biomass could be harvested sustainably in large quantities in the world, which represents a very important part of the lignocellulosic feedstock supply mix for second-generation bioethanol production [8]. As the feedstock for cellulosic ethanol production, woody biomass has significant advantages over herbaceous biomass in terms of production, harvesting, storage and transportation [9]. However, it showed greater recalcitrance than herbaceous biomass against microbial and enzymatic actions due to its tough and strong structure and high lignin content [9]. Hence, an efficient and eco-friendly pretreatment method used for overcoming the recalcitrance structure of woody biomass is required to assist the liberation of more fermentable sugars.

Various pretreatment including physical, chemical and biological approaches have been employed to disrupt the recalcitrance of lignin structure of biomass and allow the accessibility of the enzymes to polysaccharides for hydrolysis [10, 11]. Among these, biological pretreatment with microorganisms has attracted extensive attention due to its low energy consumption, environmental friendliness and less fermentation inhibitors produced [12]. White-rot basidiomycete fungi, such as *Phanerochaete chrysosporium*, *Irpex lacteus* and *Trametes versicolor* were the extensively studied strains used for woody biomass pretreatment owing to that they preferentially degraded lignin, but largely remained polysaccharides for subsequent saccharification [13–15]. The pretreatment efficiencies on woody biomass varied greatly in different white-rot fungi because of their distinguished native lignocellulose degradation pattern [16]. Despite certain degree of improvement for saccharification from woody biomass pretreated by those white-rot fungi were observed. The drawbacks of long pretreatment times, carbohydrates loss and low hydrolysis rates severely impeded its industrial application [17, 18]. Therefore, the exploration of the potential fungi strains used for woody biomass pretreatment with high efficient digestibility as well as less processing time would greatly promote the development of biorefinery industry.

*Peniophora* species strain belongs to white-rot basidiomycete has been found with impressive capacity to produce ligninolytic enzymes. For example, a strain of *Peniophora incarnate* KUC8836 has been reported with strong capacity to degrade polycyclic aromatic hydrocarbons, which ascribed to its high activities of laccase and manganese peroxidase [19]. Meanwhile, a new manganese peroxidase gene from *P*. *incarnate* KUC8836 has been overexpressed and characterized with high activity toward aromatic compound degradation [20]. Moreover, the employment of *P*. *incarnate* KUC8836 on aromatics degradation showed satisfactory effects within two weeks treatment [19, 21, 22]. These suggested that *Peniophora* species strain might be a promising candidate for woody biomass pretreatment.

In this study, a newly isolated *P*. *incarnate* strain T-7 was investigated for poplar wood pretreatment. Firstly, components analysis as well as enzymatic saccharification of pretreated poplar wood was carried out to evaluate the pretreatment efficiency of *P*. *incarnate* T-7 by submerged fermentation within a week. Subsequently, Fourier transform infrared spectroscopy (FTIR), scanning electron microscope (SEM), X-ray diffraction (XRD) and pyrolysis gas chromatography–mass spectrometry (Py-GC/MS) were conducted to deeply characterize the chemical structure change of poplar wood treated by *P*. *incarnate* T-7. Furthermore, quantitative proteomic analysis was carried out to gain mechanical insight into the selective degradation of lignocellulose components by *P*. *incarnate* T-7.

## Results and discussion

### Ligninolytic strain isolation and identification

**Fig. 1:**
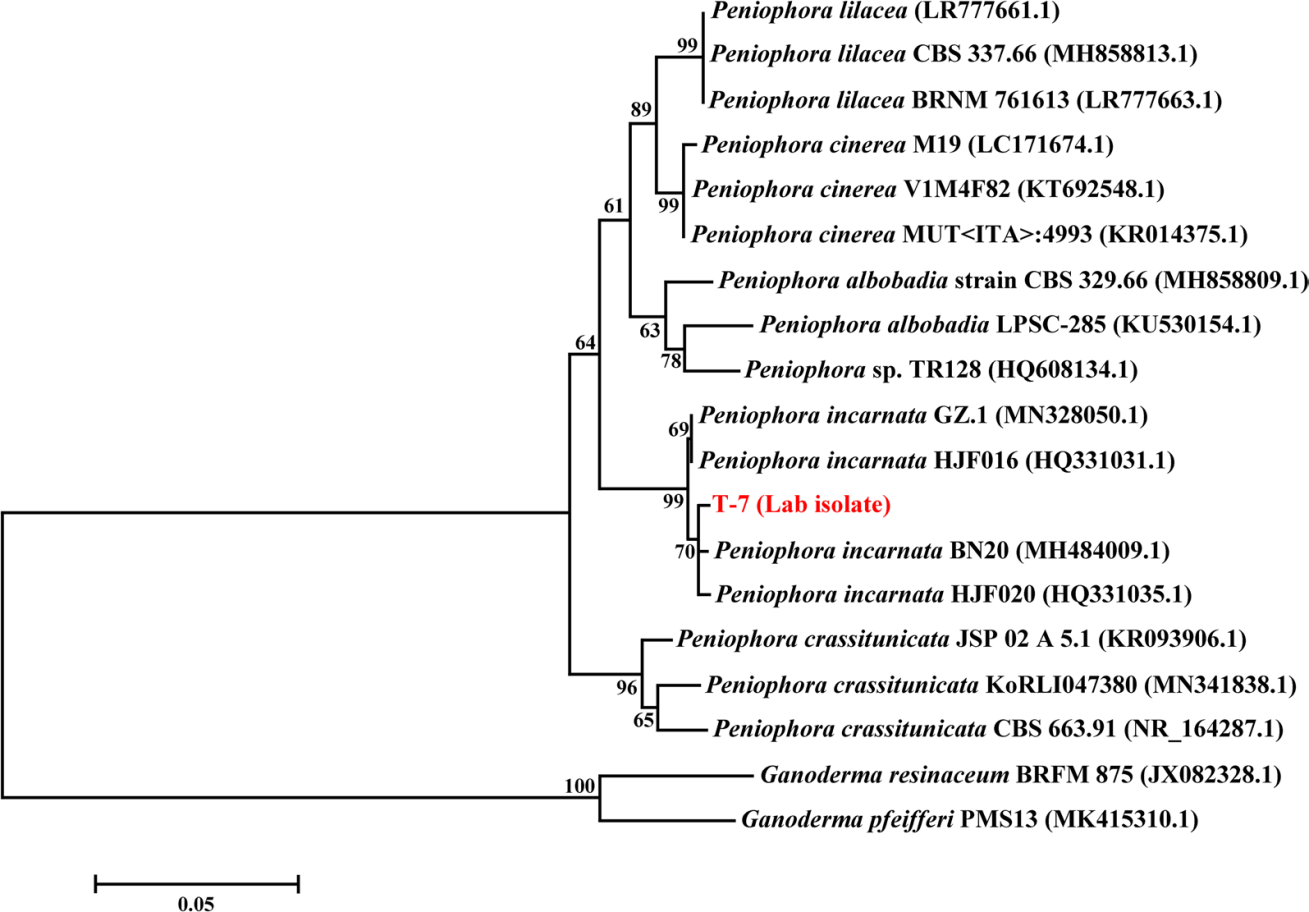
Phylogenetic analysis of strain T-7 based on ITS region sequences. The phylogenetic tree was constructed by the neighbor-joining algorithm (1000 bootstrap trials)

A total of 10 fungi strains were isolated from collected rotten wood samples. During the screening process, strain T-7 showed strong ability to utilize both guaiacol and Azure B substrates (Additional file 1: Figure S1). This suggested that strain T-7 might possess prominent capacity to produce lignin-degrading enzymes and depolymerize lignin. Subsequently, the ITS region fragment of strain T-7 was amplified and sequenced (GenBank: MT995078), which exhibited 99% similarity to *Peniophora incarnate* BN20 according to NCBI BLAST algorithm analysis. A constructed phylogenetic tree based on ITS region sequences showed that the strain fell within the *P*. *incarnate* species (Fig. 1). Consequently, strain T-7 was identified as a *P*. *incarnate* strain. This strain has since been deposited in the China General Microbiological Culture Collection (CGMCC No. 18567). A number of *P*. *incarnate* strains with strong ligninolytic activities have been studied for ligninolytic enzymes production and aromatics degradation, suggesting their great potential application on biomass pretreatment [19–21]. However, the employment of this species strains for biomass pretreatment was rarely reported.

### Characterization of *P*. *incarnate* T-7 decomposition on poplar wood

**Fig. 2:**
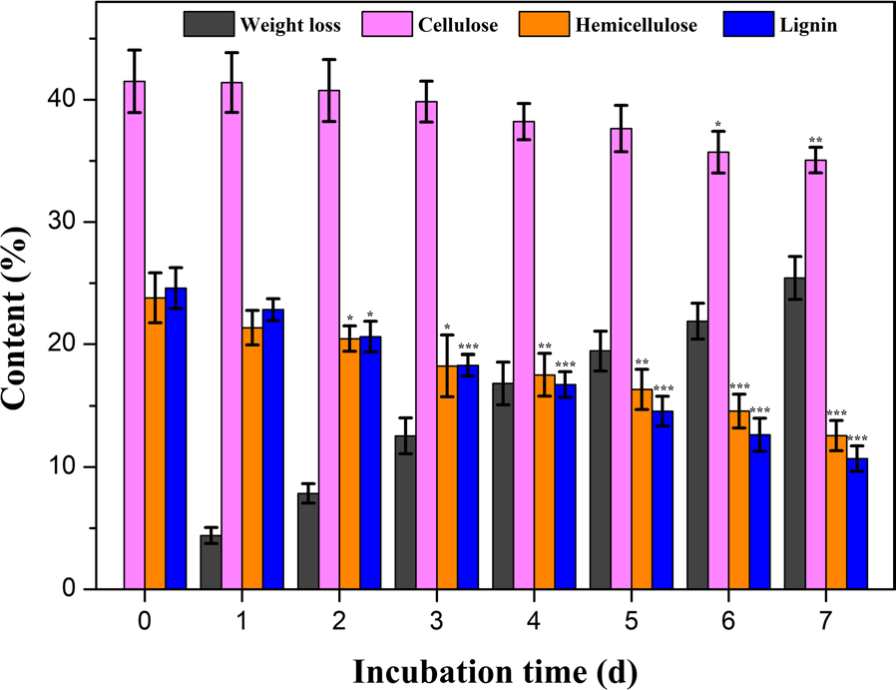
The variations weight loss and contents of cellulose, hemicellulose and lignin from poplar wood before and after treated by *P*. *incarnate* T-7 with different time. Asterisks indicate statistically significant differences from the untreated group (*P < 0.05, **P < 0.01, ***P < 0.001)

To evaluate the chemical component change of poplar wood after treated by *P*. *incarnate* T-7 in submerged fermentation, the variations of weight loss and the contents of cellulose, hemicellulose and lignin were analyzed. As shown in Fig. 2, the weight loss increased with the increment of incubation time, and the maximum weight loss reached 25.4% after seven days treatment by *P*. *incarnate* T-7. It could be deduced that the diminution of biomass weight was primarily the result of metabolism and growth of fungus. The components analysis showed that both lignin and polysaccharides continuously declined during the treatment time period, indicating that lignin, hemicellulose and cellulose were simultaneously degraded by *P*. *incarnate* T-7.

The cellulose content changed slightly during the initial five days treatment, and a significant decrease was observed in the last two days treatment relative to untreated sample. While the contents of hemicellulose and lignin showed obvious decline from day 2. After seven days treatment with *P*. *incarnate* T-7, cellulose, hemicellulose and lignin components of poplar wood decreased from 41.5%, 23.8% and 24.6% to 35.0%, 12.5% and 10.6%, respectively, with a removal of 16% cellulose, 48% hemicellulose and 70% lignin. This suggested that *P*. *incarnate* T-7 preferentially degraded lignin component of poplar wood and left large part of cellulose, which might be avail for the subsequent enzymatic hydrolysis to obtain more sugars. These results were also in line with the previous reports about the characteristics of white-rot basidiomycetes for lignocellulose degradation [17].

### Effect of *P*. *incarnate* T-7 treatment on enzymatic saccharification of poplar wood

**Fig. 3:**
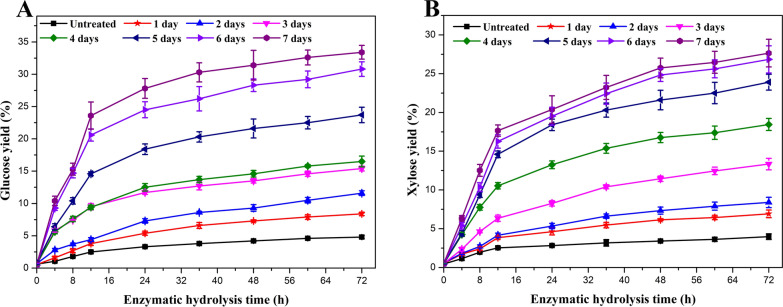
Effects of *P*. *incarnate* T-7 pretreatment on yields of glucose (**A**) and xylose (**B**) from poplar wood samples by enzymatic hydrolysis

To investigate the effect of treatment by *P*. *incarnate* T-7 on saccharification of poplar wood, enzymatic hydrolysis experiment was carried out. As shown in Fig. 3, compared to untreated group, the yields of glucose and xylose rapidly increased with the extension of treatment time. The maximum yields of glucose and xylose were up to 33.4% and 27.6%, respectively, both of which were enhanced by sevenfold relative to untreated group. According to the above compositional analysis results, the reduction in lignin was highly correlated with the yields of glucose and xylose during the treatment time. It could be speculated that the improvement of sugar yield in enzymatic hydrolysis mainly ascribed to the removal of lignin and the structural breakdown of the cellulose and hemicellulose by *P*. *incarnate* T-7. These results highlighted that the sugar yield of poplar wood obtained from enzymatic hydrolysis was greatly enhanced by the selective delignification of *P*. *incarnate* T-7. However, it should be noted that the autoclaving treatment on poplar wood during the medium preparing period might more or less contribute to promote the destruction of poplar wood structure by the strain.

Unlike agricultural biomass, woody biomass was proved to be more recalcitrant to microbial deconstruction, due to that it is structurally denser and contains higher content of lignin component than those of agricultural biomass [9]. As summarized in Table 1, the maximum sugar yield from *P*. *incarnate* T-7 treated poplar wood was only a little less than that of *Echinodontium taxodii* 2538 treated Chinese willow upon comparison to those fungi strains pretreated woody biomass. Moreover, it only took seven days to achieve the high yield of sugar in enzymatic hydrolysis by *P*. *incarnate* T-7 submerged fermentation pretreatment, which was far less than that of compared fungi strains. Several weeks to months times were taken for solid-state fermentation pretreatment by microorganisms to break down the recalcitrance lignocellulosic structure of woody biomass, which severely impeded the commercial application of biological pretreatment strategy on biorefinery industry [26]. The less processing time by *P*. *incarnate* T-7 pretreatment with high sugar yield from woody biomass showed promising potential for industrial application. Further studies focusing on operational feasibility and cost reduction are needed to aid in improvement of the techno-economic efficiency of this pretreatment for its application on biorefinery industry in the future.

**Table 1:** Sugar yields from various woody biomass pretreated by different fungi strains after enzymatic hydrolysis

Microorganism	Woody biomass	Sugar yield (%)	Pretreatment time (d)	References
*Trametes versicolor* ATCC 20,869	Rubberwood	16	90	[15]
*Phanerochaete chrysosporium* ME446	Beech wood	9.5	28	[13]
*Lenzites betulina* C5617	Poplar wood	16	28	[23]
*Trametes versicolor* C6915	Sacrau poplar	28	112	[23]
*Stereum hirsutum* KFRI234	Pine densiflora	21	56	[24]
*Irpex lacteus*	Olive tree	31	45	[14]
*Echinodontium taxodii* 2538	Chinese willow	35	119	[25]
*Echinodontium taxodii* 2538	China fir	18	119	[25]
*Peniophora incarnate* T-7	Poplar wood	33.4	7	This study

### Fourier transform infrared spectroscopy (FTIR) analysis

**Fig. 4:**
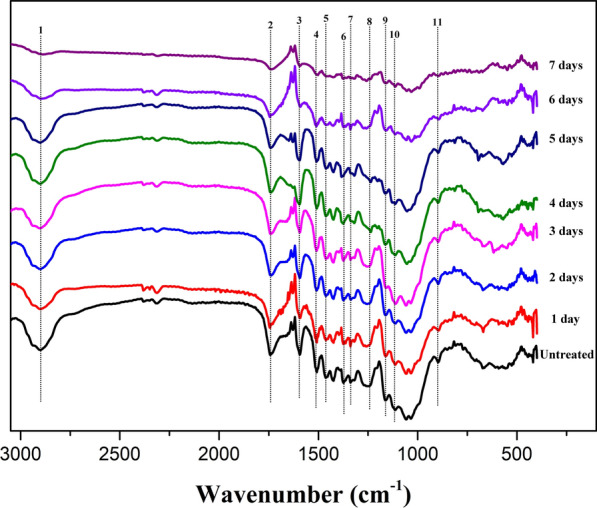
FTIR spectra of poplar wood before and after treated by *P*. *incarnate* T-7 with different time

To explore the functional group change of lignocellulose structure in poplar wood before and after *P*. *incarnate* T-7 treatment, FTIR analysis was carried out. As shown in Fig. 4, the FTIR spectra of the poplar wood samples changed significantly with the increasing treatment time by *P*. *incarnate* T-7. The characteristic peaks of FTIR spectra in cellulose, hemicellulose and lignin components from poplar wood are listed in Table 2, which were assigned based on the previous literature reports [14, 27, 28].

**Table 2:** FTIR spectra peaks assignment from poplar wood samples

Number	Wavenumber (cm^−1^)	Assignment of the peak
1	2920	Aliphatic C–H stretching vibration
2	1735	Characteristic peaks of the hemicellulose–lignin complex
3	1605	Aromatic skeleton vibration in lignin
4	1510	Guaiacyl ring from lignin
5	1430	Cellulose I absorption band
6	1375	C–H deformation of crystalline cellulose and hemicellulose
7	1320	Aromatic ring breathing syringyl and guaiacyl units
8	1250	Hemicellulose peaks (acetyl groups C-O)
9	1162	C–O–C vibration in cellulose and hemicellulose
10	1105	C–OH skeletal vibration of cellulose
11	898	C–O–C stretching of amorphous cellulose

The intensities of bands at 1605 cm^−1^, 1510 cm^−1^ and 1320 cm^−1^ which were assigned to aromatic skeleton vibration, guaiacyl ring unit and C–O vibration of syringyl unit in lignin structure, respectively, showed general descending trends during the incubation period by *P*. *incarnate* T-7, and the most pronounced declines were observed in day 7. This clearly suggested that lignin component was substantially destructed by the strain across the treatment time. Declines of peaks intensities with different extent observed at 1430 cm^−1^ (cellulose I absorption band), 1375 cm^−1^ (C–H deformation of crystalline cellulose and hemicellulose), 1250 cm^−1^ (hemicellulose peaks), 1162 cm^−1^ (C–O–C vibration in cellulose and hemicellulose), 1105 cm^−1^ (C–OH skeletal vibration of cellulose) and 898 cm^−1^ (C–O–C stretching of amorphous cellulose) of from poplar wood after treated by *P*. *incarnate* T-7 suggested that both cellulose and hemicellulose components were degraded during the incubation time. Interestingly, most of those peaks intensities, especially the peaks at 1375 cm^−1^, 1105 cm^−1^ and 898 cm^−1^ decreased slightly during the initial five days incubation, and significant diminutions were observed in the last two days treatment. These implied that *P*. *incarnate* T-7 mainly attacked lignin moiety with less cellulose degradation during the early stage of treatment, and cellulose especially crystalline region cellulose might be severely deconstructed from the last two days treatment. This was in contrasted with the study of Xu et al. that cellulose and hemicellulose were dominantly degraded in the early stage of *Irpex lacteus* CD2 pretreatment, followed by lignin degradation [29]. Though preferential degrading lignin relative to polysaccharides was the characteristic of white-rot fungi, the degradation patterns varied among those fungi, which mainly depended on the species of fungus strain, kinds of feedstocks and fermentation ways [17]. In this study, the continuous liberated amount of pentose from the hemicellulose degradation during the early stage of treatment could support the growth of fungus, which might repress a part of cellulose degrading enzymes expression [30]. Hence, cellulose component was less degraded in the early stage of treatment. The breakdown of cellulose crystalline structure in the last period of treatment could potentially render more accessibility of enzyme in saccharification process, which was corresponding with the enzymatic saccharification results that the sugar yields of poplar wood samples from the last two days treatment were obviously higher than those of early stage of treatment.

### Scanning electron microscope (SEM) analysis

**Fig. 5:**
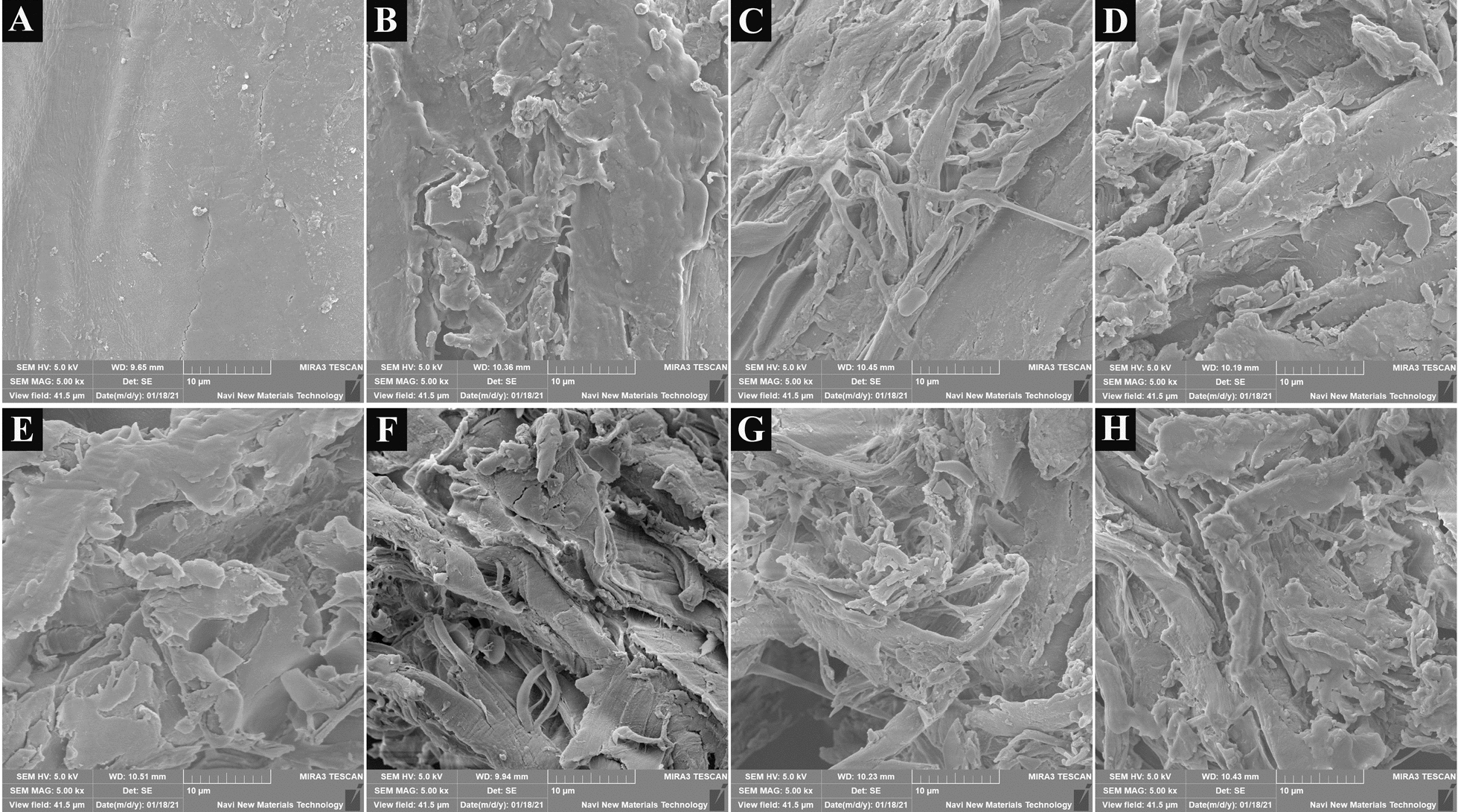
SEM images of poplar wood before (**A**) and after treated by *P*. *incarnate* T-7 with different time (**B–H**). **B–H** represent poplar wood treated by the fungus for 1–7 days, respectively

To evaluate the effect of *P*. *incarnate* T-7 treatment on surface microstructure morphology of poplar wood, SEM analysis was carried out. As shown in Fig. 5, the surface of untreated poplar wood showed a smooth flat and compact structure. However, the surface structure of poplar wood after *P*. *incarnate* T-7 treatment was significantly different with that of untreated sample, which was severely modified by the fungus with more rugged and loose porous structures. In addition, more and more fiber bundles from poplar wood were exposed out with prolong of the treatment time. After seven days treatment, the surface structure of poplar wood was nearly destructed by the fungus with lots of fiber bundles observed. These indicated that the poplar wood structure was strongly broken down after *P*. *incarnate* T-7 treatment, which was in line with the results of FTIR analysis.

### X-ray diffraction (XRD) analysis

The crystalline index of cellulose is a crucial factor representing the relative proportion of the cellulose crystalline region which was considered as the major recalcitrance region toward enzymatic hydrolysis [31]. To evaluate the changes of cellulose crystalline structure of poplar wood before and after *P*. *incarnate* T-7 treatment, XRD analysis was carried out.

**Fig. 6:**
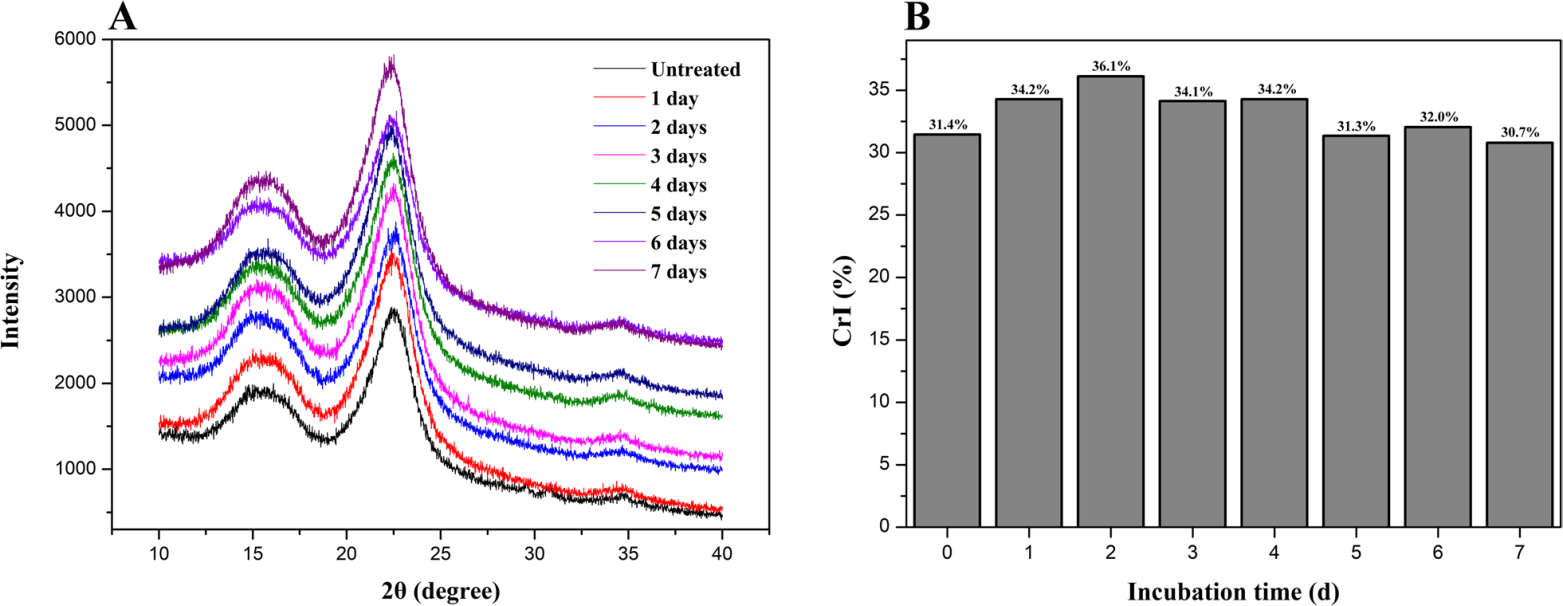
X-ray diffraction spectra (**A**) and cellulose crystalline index (CrI) (**B**) of poplar wood before and after treated by *P*. *incarnate* T-7 with different time

As shown in Fig. 6A, two peaks at 2θ = 22.3 and 2θ = 16.2 assigned to the crystallographic plane and amorphous phase in cellulose structure, respectively, were observed in all the detected samples. However, the intensities of these two peaks in *P*. *incarnate* T-7 treated samples showed different extent increase relative to untreated sample. As shown in Fig. 6B, compared to untreated sample, the crystalline index (CrI) value of cellulose in poplar wood exhibited a near increment trend during the initial four days of *P*. *incarnate* T-7 treatment, with the maximum value of 36.1% at second day. After that, it showed a general decline tendency in the later period, with the minimum value of 30.7% at 7th day. The augment of CrI values in *P*. *incarnate* T-7 treated samples during the initial four days might be resulted by the destruction of the amorphous components in poplar wood, such as lignin and hemicellulose [32], while the reduction of CrI values of poplar wood samples from the last stage of treatment could be caused by the breakdown of the crystallographic region of the cellulose component. The destruction of crystalline cellulose structure was in favor of the cellulase accessibility, which could enhance the saccharification efficiency [33]. These were in well agreement with the above results of compositional investigation, enzymatic saccharification and FTIR analysis.

### Pyrolysis–gas chromatography/mass spectrometry (Py-GC/MS) analysis

To further explore the effect of *P*. *incarnate* T-7 treatment on lignin structure of poplar wood, Py-GC/MS analysis was carried out. The pyrograms of poplar wood samples are shown in Additional file 2: Figure S2. The species of identified pyrolysates from *P*. *incarnate* T-7 treated poplar wood was similar to that of untreated one, which were classified as three categories, i.e., syringyl (S)-, guaiacyl (G)- and *p*-hydroxyphenyl (H)-type lignin-derivatives, according to the structure analysis (Additional file 3: Table S1). However, the relative abundances of these identified compounds from lignin samples before and after treatment with fungus showed significant distinction. As listed in Table 3, the total abundance of lignin derivates showed decline trend during the treatment period by *P*. *incarnate* T-7, reducing from 22.39% in untreated sample to 11.87% after seven days treatment, which was in accordance with the compositional analysis results. This further confirmed that lignin structure of poplar wood was efficiently depolymerized by the fungus.

**Table 3:** The amounts of lignin-derived pyrolysates identified by Py-GC/MS from poplar wood samples before and after treated by *P*. *incarnate* T-7 with different time

Category	Untreated	1 d	2 d	3 d	4 d	5 d	6 d	7 d
Total peaks areas of lignin derivates	22.39	21.88	20.26	19.62	18.45	16.24	13.35	11.87
Total peak areas of H	1.74	1.92	1.31	1.83	1.52	1.88	1.51	1.64
Total peak areas of S	15.92	15.31	14.57	13.64	12.27	10.52	8.61	6.88
Total peak areas of G	4.73	4.65	4.38	4.15	4.66	3.84	3.23	3.35
S/G	3.37	3.29	3.33	3.29	2.63	2.74	2.67	2.05

After seven days treatment with fungus, the total peak areas of H-, S- and G-type lignin derivatives declined from 1.74%, 15.92% and 4.73% to 1.64%, 6.88% and 3.35%, respectively. In addition, the ratio of S/G decreased from 3.37 to 2.05. These suggested that *P*. *incarnate* T-7 could degrade those three types of lignin units from poplar wood during the submerged fermentation treatment, but it preferentially degraded S-type unit. The variations of S/G ratio were inconsistence in different studies of lignin component degradation by white-rot fungi strains. A number of studies showed that S-type unit was preferentially degraded by the fungi strains, which was similar to the result of our manuscript [34–36]. On the contrary, some studies demonstrated that G-type unit was easier to be attacked [37, 38]. It should be noted that the feedstocks, fungus strains species and fermentation types were different in those studies, which might cause the inconsistence preferential degradation for lignin units [39]. Among those lignin units, S-type unit was considered as the most recalcitrant structure against fungal delignification, since it contains two methoxyl groups with lower redox potential [40]. Meanwhile, the lignin component of poplar wood was dominantly occupied by S-type unit, which further hindered its delignification by microorganism. In this study, the efficient removal of S-type unit by *P*. *incarnate* T-7 demonstrated its prominent delignification capacity, which largely contributed to the depolymerization of poplar wood structure.

Unlike S-type unit, the total peak areas of H- and G-type lignin derivatives showed fluctuation changes, albeit they generally declined during the *P*. *incarnate* T-7 treatment. During the fungal delignification of biomass, demethoxylation was the initial and critical step for lignin degradation [41]. The substantial demethoxylation of S-type unit could lead to G- and H-type products generation. Hence, it could be deduced that parts of G- and H-type units might be derived from the S-type unit demethoxylation by *P*. *incarnate* T-7 during the treatment, which partially interfered they content variation [34].

### Transcriptomic and quantitative proteomic analysis of *P*. *incarnate* T-7 on poplar wood substrate

**Fig. 7:**
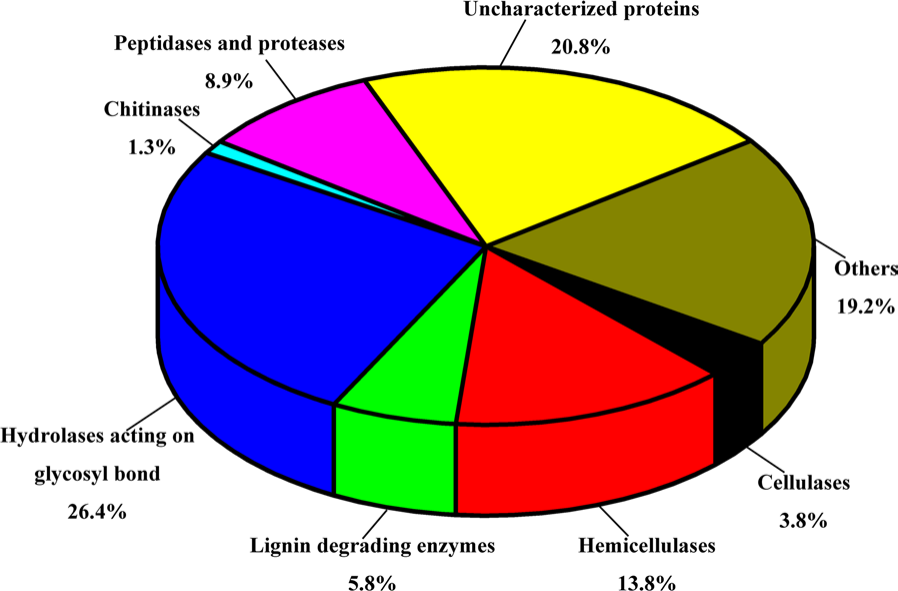
Relative contents of identified proteins based on functional classification

To explore the mechanism of selective degradation of lignocellulose components by *P*. *incarnate* T-7, transcriptomic and TMT labeling quantitative proteomic analysis was performed to explore the differential expression of lignocellulosic enzymes of *P*. *incarnate* T-7 by submerged fermentation on poplar wood substrate relative to glucose substrate. Transcriptome analysis was conducted to examine expression of genes encoding proteins on poplar wood and glucose substrate. The transcriptome results identified 1,3176 unigenes (Additional file 4), including 125 genes encoding lignin-degrading enzymes, 93 genes encoding hemicellulases, 57 genes encoding cellulases and 120 genes encoding glycoside hydrolases (Additional file 5). Among these, 53 genes were significantly upregulated in poplar wood substrate relative to glucose substrate (Additional file 6). A total of 390 proteins were identified from the secretome of *P*. *incarnate* T-7 (Additional file 7), which were functionally divided into eight categories based on their biological role (Additional file 8). As shown in Fig. 7, among the identified proteins, the total number of lignocellulolytic enzymes including lignin-degrading enzymes, hemicellulases, cellulases and hydrolases acting on glycosyl bond together formed the largest group of proteins, which comprised 5.8%, 13.8%, 3.8% and 26.4%, respectively. In addition, the activities of lignocellulolytic enzymes, including endoglucanase, exoglucanase, β-glucosidase, xylanase, lignin peroxidase, manganese peroxidase and laccase were all detected during the *P*. *incarnate* T-7 fermentation (Additional file 9: Figure S3). These indicated the prowess lignocellulolytic enzymes cocktail of *P*. *incarnate* T-7.

**Fig. 8:**
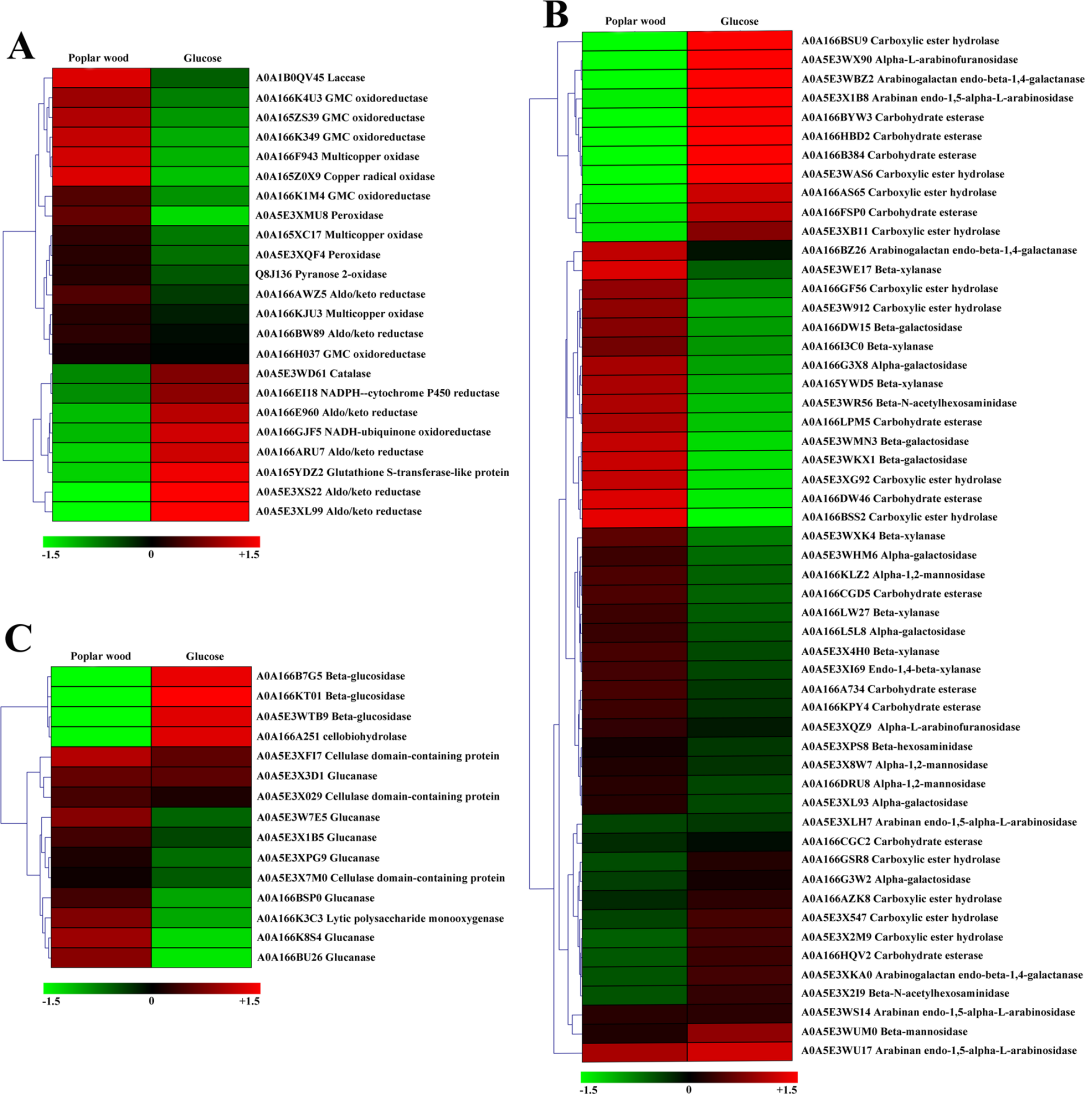
Hierarchical clustering of main lignocellulolytic enzymes including lignin-degrading enzymes (**A**), hemicellulases (**B**) and cellulases (**C**) by *P*. *incarnate* T-7 with different expression levels when incubated on glucose and poplar wood substrates, respectively

There were in total 23 lignin-degrading enzymes identified from the secretory proteins of *P*. *incarnate* T-7 (Fig. 8A). The significant groups of lignin-degrading enzymes, including laccase, multicopper oxidase, peroxidase and other auxiliary enzymes such as copper radical oxidase and GMC oxidoreductase which provided hydrogen peroxidase for lignin-degrading peroxidases were found [42, 43]. Although the canonical lignin peroxidase and manganese peroxidase genes were absence of according to transcriptomic analysis, and these two proteins were also unidentified in secretomic results. There were in total 16 genes encoding peroxidase (class II) identified in transcriptome results. Meanwhile, two peroxidases were detected in the secretory proteins of *P*. *incarnate* T-7. Given that both lignin peroxidase and manganese peroxidase belong to class II peroxidase, the identified peroxidase genes might function as these two enzymes [44]. Further studies are needed to characterize the function of those peroxidases in *P*. *incarnate* T-7 during lignin degradation. In addition, catalase has been reported to participate in lignin degradation, the role of which was similar to those of lignin peroxidase and manganese peroxidase [45]. The presence of this protein in secretome of *P*. *incarnate* T-7 might also play important role during degradation of poplar wood lignin component. In addition, most of those identified lignin-degrading enzymes showed higher expression level on poplar wood substrate compared to glucose substrate (Fig. 8A), which was consistence with the lignin-degrading enzymes activities detected result (Additional file 9: Figure S3A, B and C). Transcriptome results also revealed that the expression levels of two laccase genes and one multicopper oxidase gene were highly upregulated on poplar wood substrate relative glucose substrate (Additional file 10: Figure S4A). These indicated the great lignin degradation capacity of *P*. *incarnate* T-7, which was in line with the above results that a large amount of lignin component from poplar wood was removed by the fungus.

It was noticeable that there were 54 proteins involved in hemicellulose degradation identified in secretome of *P*. *incarnate* T-7, which was higher than that of cellulase and lignin-degrading enzymes (Fig. 8B). These contained six carbohydrate esterases, nine carboxylic ester hydrolases, three beta-xylanses and four mannosidases which acted on the major components of hemicellulose, and other auxiliary enzymes which synergistically break side chains of hemicellulose, including four arabinosidases, two arabinofuranosidases and eight galactosidases [46]. The existence of abundant hemicellulases in secretome of *P*. *incarnate* T-7 implied its potential strong hemicellulose degradation ability. Moreover, compared with glucose substrate, the expression levels of majority of those hemicellulases were highly upregulated on poplar wood substrate (Fig. 8B), which was in line with the results of transcriptome analysis (Additional file 10: Figure S4B) and xylanase activity in these two substrates (Additional file 9: Figure S3D). These results explained that almost half of hemicellulose was removed from poplar wood after *P*. *incarnate* T-7 treatment.

The number of identified cellulases was only 15, which was the least among these lignocellulolytic enzymes (Fig. 8C). Among those, the typical cellulase such as endoglucanase and exoglucanase was undetected. These results potentially suggested its limited cellulose degradation capacity, which was consistent with the results that cellulose component was less lost by the *P*. *incarnate* T-7 treatment. Interestingly, a lytic polysaccharide monooxygenase (LPMO) was detected in secretome of this strain. LPMO has been found with the capacity to degrade crystalline region cellulose by oxidative cleavage, which could significantly promote crystalline cellulose deconstruction [47]. In addition, the protein expression level of LPMO was significantly higher on poplar wood substrate than that of glucose substrate (Fig. 8C), which was in accordance with transcriptomic analysis result (Additional file 10: Figure S4C). Hence, the decline of crystalline index of cellulose from poplar wood after treated by *P*. *incarnate* T-7 might be partially ascribed to the catalytic action of this LPMO.

## Conclusions

In this study, a new white-rot basidiomycete *P*. *incarnate* T-7 was isolated for poplar wood pretreatment by submerged fermentation. The componential analysis revealed that lignin was the major component removed from poplar wood by *P*. *incarnate* T-7 during the submerged fermentation, with less cellulose component lost. Enzymatic saccharification analysis showed that the maximum yields of glucose and xylose reached 33.4% and 27.6%, respectively, from poplar wood after seven days treatment by the strain, both of which were improved by sevenfold relative to untreated group. Chemical structure characterization including FTIR, SEM, XRD and Py-GC/MS analyses confirmed that the lignocellulosic structure of poplar wood was severely broken down by *P*. *incarnate* T-7, and it preferentially degraded lignin component. Proteomic analysis demonstrated that *P*. *incarnate* T-7 could secrete a considerable amount of lignocellulolytic enzymes, especially with abundant lignin-degrading enzymes and hemicellulases. Both proteome and transcriptome data revealed that most of those lignocellulolytic enzymes were highly upregulated on poplar wood substrate relative to glucose substrate. This study demonstrated that the enzymatic saccharification efficiency of poplar wood could be greatly enhanced by *P*. *incarnate* T-7 via submerged fermentation with short time of seven days, which showed a promising prospect applying on industrial woody biomass biorefinery.

## Methods

### Materials

Air-dried poplar wood (*Populus tomentosa* Carr.) was collected in the Yueyang city, Hunan province, China. It was cut into chips and was subsequently milled to pass through a 20-mesh screen and stored under dried conditions until use.

### Isolation and identification of lignin-degrading fungal strain

Samples of rotten wood were collected from the forests of Yuelu mountain in Changsha city, Hunan province, China. After washing three times with sterile water, the sample was placed in the center of Petri dish containing potato dextrose agar (PDA) medium (potato extract 200 g/L, glucose 10 g/L, MgSO_4_ 1.5 g/L, KH_2_PO_4_ 3.0 g/L, vitamin B 0.05 g/L, agar 15 g/L), and the plate was incubated at 28 °C. The fungal strains growing out around the rotten wood sample were isolated to new plates and cultivated for 2 to 3 generations, respectively. The pure isolates were inoculated in selective medium for ligninolytic strain screening. The selective medium included Azure B-containing and guaiacol-containing PDA medium (potato extract 200 g/L, Azure B or guaiacol 0.1 g/L, glucose 10 g/L, MgSO_4_ 1.5 g/L, KH_2_PO_4_ 3.0 g/L, vitamin B 0.05 g/L, agar 15 g/L). The isolates produced the largest diameter of the decolorization zone in Azure B-containing PDA medium or colorization zone in guaiacol-containing PDA medium were selected for further study.

Morphological characterization and internal transcribed region sequence (ITS) analysis were employed to identify the fungus strain. The morphological features of fungal strain were determined by investigation of the colonies and mycelium. Total DNA of fungus strain was extracted with a Fungi Genomic DNA Extraction Kit (Solarbio, Beijing, China). Amplication of ITS region sequence was performed using ITS 1 forward (TCCGTAGGTG AACCTGCGG) and ITS 4 reverse (TCCTCCGCTTATTGATATGC) as the primer. The amplified products were sequenced and analyzed by the NCBI BLAST algorithm tool. The phylogenetic analysis was conducted by MEGA 5.0 based on neighbor-joining algorithm.

### Pretreatment of poplar wood with fungal strain by submerged fermentation

The fully grown fungus mycelia on PDA medium were scraped off into basic medium (glucose 20 g/L, peptone 1 g/L, yeast extract 2 g/L, KH_2_PO_4_ 4 g/L, (NH_4_)_2_SO_4_ 4 g/L, CaCl_2_ 0.3 g/L, MgSO_4_ 0.3 g/L, NaCl 2 g/L) and incubated at 28 °C for three days with shaking at 200 rpm. The prepared cultures were used as inoculum for submerged fermentation. Two grams of ground poplar wood powder with 100 mL of mineral salt solution (KH_2_PO_4_ 4 g/L, (NH_4_)_2_SO_4_ 4 g/L, CaCl_2_ 0.3 g/L, MgSO_4_ 0.3 g/L, MnSO_4_ 0.0016 g/L, CoCl_2_ 0.002 g/L) were added to 250-mL Erlenmeyer flasks. The flasks were autoclaved at 121 °C for 30 min. Each flask was inoculated with 5 mL of the prepared suspension and incubated at 200 rpm and 28 °C for 7 days. Sterilized non-inoculated medium was used as a control. Samples were withdrawn at every 24-h interval for the following analysis.

### Enzymatic saccharification

Enzymatic saccharification was carried out in conical flasks containing 5% (w/v) poplar wood substrate, 50 mM sodium citrate buffer (pH 4.8), 20 FPU/g substrate of cellulase and 0.01% sodium azide at 50 °C, 150 rpm for 72 h. Samples of the enzymatic hydrolysates were withdrawn at different time points for released sugar analysis. High-performance liquid chromatography (HPLC) system equipped with an Aminex HPX-87H column (300 mm × 7.8 mm) was employed for determination of hydrolyzed glucose and xylose concentrations. The glucose or xylose yield from substrate hydrolysis was calculated as the following formula:

$$\rm{Glucose\,yield}\left( \%  \right) = \left( {{m_{gh}}/{m_{go}}} \right) \times 0.9 \times 100$$

$$\rm{Xylose\,yield}\left( \%  \right) = \left( {{m_{xh}}/{m_{xo}}} \right) \times 0.88 \times 100$$

where m_gh_ and m_xh_ represent the amount of released glucose and xylose, respectively, and mg_0_ and m_x0_ represent the amount of cellulose and xylose from poplar wood substrate, respectively

### Enzyme activity assay

The culture samples of submerged fermentation were harvested at every 24-h interval and centrifuged at 1,2000 rpm, 4 °C for 10 min, and the supernatants were collected for enzyme activity assay. Activities of endoglucanase, exoglucanase, β-glucosidase, xylanase, lignin peroxidase, manganese peroxidase and laccase were determined as previously described [45].

### Secretory protein extraction

The cultures of the fungus strains grew in poplar wood and glucose substrates containing media, respectively, were collected at 4th day. After filtering, the collected cultures were centrifuged at 4000 rpm, 4 °C for 10 min, and the supernatant was further filtered through 0.45 μm membranes. Ammonium sulfate precipitation was employed for concentrating the supernatant protein fraction. The precipitated protein pellets were redissolved in an appropriate volume of the preparation solution buffer (8 M urea, 4% (w/v) 3-[(3-cholamidopropyl) dimethylammonio]-1-propanesulfonate (CHAPS) and 40 mM dithiothreitol). Protein concentration was assayed using a non-interference protein assay kit (Sangon Biotech, Shanghai, China).

### Tandem mass tag (TMT) labeling quantitative proteomic analysis

The quantitative analysis of proteomic protein was conducted using TMT labeling method. The protein digestion was performed as described by Hou et al. [48]. Briefly, dithiothreitol was firstly added into 100 μg of extracted protein with 25 mM final concentration and incubated at 37 °C for 1 h. Then, iodoacetamide with 50 mM final concentration was added and incubated in darkness at room temperature for 30 min. After that, the protein solution was precipitated by ice-cold acetone and the collected pellets were dissolved in 100 mM triethylammonium bicarbonate. Subsequently, sequencing grade trypsin was added at a protein/trypsin ratio of 50:1 (w/w) and incubated at 37 °C for 16 h. The digested peptides were labeled with a TMT label reagent kit at room temperature for 1 h, and formic acid was added with 1% final concentration to quench the labeling reaction. The labeled samples were pooled in equal proportion and subjected to nano-liquid chromatography–tandem mass spectrometry (nanoLC–MS/MS). NanoLC–MS/MS analysis was performed on an Orbitrap Fusion Tribrid MS (Thermo, Waltham, MA) equipped with an UltiMate 3000 system according to the procedure described by Hou et al. [48]. The collected raw MS/MS data were processed using Maxquant proteomics software (Version 1.6.4) against UniProt proteomes of *Peniophora* genus. The tolerances of peptide mass and fragment mass were set as ± 10 ppm and ± 0.01 Da, respectively. The desired false discovery rate (FDR) was set to 1% for validation of peptide and protein identification. The mass spectrometry proteomics data have been deposited in the ProteomeXchange Consortium via the PRIDE partner repository with the dataset identifier PXD025248 [49].

### RNA extraction and transcriptome sequencing

The mycelia of fungus from poplar wood and glucose substrates containing media were collected at 4th day for RNA extraction. Total RNA was extracted using TRIzol reagent (Invitrogen, Carlsbad, CA, USA). The purity and integrity of the total RNA were assessed using the NanoPhotometer spectrophotometer (IMPLEN, CA, USA) and RNA Nano 6000 Assay Kit of the Bioanalyzer 2100 system (Agilent Technologies, CA, USA), respectively. RNA sequencing libraries were prepared by NEBNext UltraTM RNA Library Prep Kit for Illumina (NEB, USA) following manufacturer’s instructions, and index codes were added to attribute sequences to each sample. The clustering of the index-coded samples was performed on a cBot Cluster Generation System using TruSeq PE Cluster Kit v3-cBot-HS (Illumia). After cluster generation, the library preparations were sequenced at Novogene Bioinformatics Technology Co., LTD (Beijing, China) using an Illumina Novaseq platform with 150-bp paired-end reads generated. The sequenced raw data were filtered to remove low-quality reads and adapter and poly-N containing-reads. Unigene annotation and function was performed by BLAST algorithm using the following seven databases: NCBI non-redundant protein sequences (Nr), NCBI non-redundant nucleotide sequences (Nt), protein family (Pfam), clusters of orthologous groups of protein (COG), KEGG Ortholog database (KO), Gene Ontology (GO) and Swiss-Prot.

The gene expression levels were calculated using the fragments per kilobase of exon per million fragments mapped (FPKM) method [50]. The significant differential expression of read counts was conducted by DESeq2 [51] with a *P* < 0.05 and a false discovery rate (FDR) < 0.01.

### Chemical analysis of from poplar wood components

The components of poplar wood including cellulose, hemicellulose and lignin were analyzed according to the National Renewable Energy Laboratory (NREL, Colorado, USA) procedure for biomass [52]. The degradation efficiencies of poplar wood biomass, cellulose, hemicellulose and lignin were calculated by the following formula [53]:

Biomass (cellulose, hemicellulose, lignin) loss (%) = [(m_0_-m_n_)/m_0_] × 100,where m_0_ represents the initial poplar wood weight or (cellulose, hemicellulose, lignin) content in the sterilized medium; m_n_ represents the nth day processing poplar wood weight or (cellulose, hemicellulose, lignin) content.

### Fourier transformation infrared spectroscopy (FTIR) analysis

The functional groups of samples were investigated by FTIR analysis that conducted on a Varian 2000 FTIR spectrometer. For each sample, FTIR spectra were acquired in the range from 4000 cm^−1^ to 400 cm^−1^ by taking 32 scans at 4 cm^−1^ resolution.

### Scanning electron microscope (SEM) analysis

SEM analysis was carried out with a MIRA3 LMH scanning electron microscopy (TESCAN, Czech Republic). The samples were covered with gold to prevent buildup of static charges.

### X-ray diffractometer (XRD) analysis

The cellulose crystallinity of samples was measured by a Bruker D8 Advance powder XRD instrument equipped with a Cu Kα source tube. The XRD spectra were recorded from the angular 2θ of 15° to 80° at the speed of 2°/min. The cellulose crystalline index (CrI) of samples was calculated according to the method of Park et al. [54] as following formula:

$$CrI{\text{ }}\left( \%  \right) = \left[ {\left( {{I_{002}} - {I_{am}}} \right)/{I_{002}}} \right] \times 100$$

where I_002_ represents the maximum intensity at angular 2θ of 22.3°, and Iam represents the minimum intensity at angular 2θ of 16.2°.

### Pyrolysis gas chromatography–mass spectrometry (Py-GC/MS) analysis

Analytical pyrolysis of from poplar wood was performed using a EGA/PY-3030D pyrolyzer coupled to a gas chromatography with mass spectrometric detection. The procedures for pyrolysis and GC–MS analysis were conducted as previously described [53]. The mass spectra of pyrolysis products were identified by the National Institute of Standards and Technology (NIST) mass spectrum library. The relative abundances of lignin-derived pyrolysis products were calculated as previously described [53].

## Supplementary Information

**Additional file 1: Figure S1.** Qualitative detection of *P*. *incarnate* T-7 degradation ability for guaiacol and Azure B substrates. **A**, **B**, guaiacol-containing PDA medium; C and D, Azure B-containing PDA medium.

**Additional file 2: Figure S2.** The pyrograms of poplar wood samples before and after treated by *P*. *incarnate* T-7 with different time.

**Additional file 3: Table S1.** The lignin-derived pyrolysates from poplar wood samples before and after treated by *P*. *incarnate* T-7 with different time identified by Py-GC/MS with relative peak areas.

**Additional file 4: **Summary of *P*. *incarnate* T-7 unigenes annotations from transcriptomic sequencing**.**

**Additional file 5: **The annotation of lignocellulolytic enzyme genes in the transcriptome.

**Additional file 6: **Differential expression analysis of *P*. *incarnate* T-7 total genes in comparison of poplar wood substrate versus glucose substrate.

**Additional file 7: **The identified proteins from secretome of *P*. *incarnate* T-7 by submerged fermentation on poplar wood substrate and glucose substrate, respectively.

**Additional file 8: **Classification of identified proteins from secretome of *P*. *incarnate* T-7 based on biological function.

**Additional file 9: Figure S3.** The comparison of lignocellulolytic enzyme activities of *P*. *incarnate* T-7 on poplar wood substrate and glucose substrate. A-G represent activities of lignin peroxidase, manganese peroxidase, laccase, xylanase, endoglucanase and β-glucosidase, exoglucanase, respectively.

**Additional file 10: Figure S4.** Hierarchical clustering of expression of lignocellulolytic enzymes including lignin-degrading enzymes (**A**), hemicellulases (**B**) and cellulases (**C**) encoding transcripts by *P*. *incarnate* T-7 on poplar wood substrate and glucose substrate, respectively.

**Additional file 11: Table S2.** Percentage numbers of the abundant annotated species from transcriptomic sequencing.

## Data Availability

All data generated or analyzed during this study are included in this published article and its supplementary information file.

## Abbreviations

FTIR: Fourier transform infrared spectroscopy
XRD: X-ray diffraction
Py-GC/MS: Pyrolysis gas chromatography–mass spectrometry
PDA: Potato dextrose agar
ITS: Internal transcribed region sequence
HPLC: High-performance liquid chromatography
CHAPS: (3-[(3-Cholamidopropyl) dimethylammonio]-1-propanesulfonate)
TMT: Tandem mass tag
nanoLC–MS/MS: Nano-liquid chromatography–tandem mass spectrometry
FDR: False discovery rat
NIST: National Institute of Standards and Technology
CrI: Cellulose crystalline index
S: Syringyl
G: Guaiacyl
H: *p*-Hydroxyphenyl
LPMO: Lytic polysaccharide monooxygenase

## References

[1] Chundawat SP, Beckham GT, Himmel ME, Dale BE (2011). Deconstruction of lignocellulosic biomass to fuels and chemicals. Annu Rev Chem Biomol Eng.

[2] Menon V, Rao M (2012). Trends in bioconversion of lignocellulose: Biofuels, platform chemicals & biorefinery concept. Prog Energy Combust Sci.

[3] Sun Y, Cheng J (2002). Hydrolysis of lignocellulosic materials for ethanol production a review. Bioresour Technol.

[4] Mood SH, Golfeshan AH, Tabatabaei M, Jouzani GS, Najafi GH, Gholami M, Ardjmand M (2013). Lignocellulosic biomass to bioethanol, a comprehensive review with a focus on pretreatment. Renew Sust Energ Rev.

[5] Schutyser W, Renders T, Van den Bosch S, Koelewijn SF, Beckham GT, Sels BF (2018). Chemicals from lignin: an interplay of lignocellulose fractionation, depolymerisation, and upgrading. Chem Soc Rev.

[6] Xiao MZ, Chen WJ, Hong S, Pang B, Cao XF, Wang YY (2019). Structural characterization of lignin in heartwood, sapwood, and bark of eucalyptus. Int J Biol Macromol.

[7] Zeng Y, Zhao S, Yang S, Ding SY (2014). Lignin plays a negative role in the biochemical process for producing lignocellulosic biofuels. Curr Opin Biotechnol.

[8] Zhu JY, Pan X, Zalesny RS (2010). Pretreatment of woody biomass for biofuel production: energy efficiency, technologies, and recalcitrance. Appl Microbiol Biotechnol.

[9] Zhu JY, Pan XJ (2010). Woody biomass pretreatment for cellulosic ethanol production: Technology and energy consumption evaluation. Bioresour Technol.

[10] Cheah WY, Sankaran R, Show PL, Ibrahim TNB, Chew KW, Culaba A (2020). Pretreatment methods for lignocellulosic biofuels production: current advances, challenges and future prospects. Biofuel Res J.

[11] Abraham A, Mathew AK, Park H, Choi O, Sindhu R, Parameswaran B (2020). Pretreatment strategies for enhanced biogas production from lignocellulosic biomass. Bioresour Technol.

[12] Sindhu R, Binod P, Pandey A (2016). Biological pretreatment of lignocellulosic biomass-An overview. Bioresour Technol.

[13] Sawada T, Nakamura Y, Kobayashi F, Kuwahara M, Watanabe T (1995). Effects of fungal pretreatment and steam explosion pretreatment on enzymatic saccharification of plant biomass. Biotechnol Bioeng.

[14] Martínez-Patiño JC, Lu-Chau TA, Gullón B, Ruiz E, Romero I, Castro E (2018). Application of a combined fungal and diluted acid pretreatment on olive tree biomass. Ind Crops Prod.

[15] Nazarpour F, Abdullah DK, Abdullah N, Zamiri R (2013). Evaluation of biological pretreatment of rubberwood with white rot fungi for enzymatic hydrolysis. Materials.

[16] Sanchez C (2009). Lignocellulosic residues: biodegradation and bioconversion by fungi. Biotechnol Adv.

[17] Wan C, Li Y (2012). Fungal pretreatment of lignocellulosic biomass. Biotechnol Adv.

[18] Salvachua D, Prieto A, Lopez-Abelairas M, Lu-Chau T, Martinez AT, Martinez MJ (2011). Fungal pretreatment: An alternative in second-generation ethanol from wheat straw. Bioresour Technol.

[19] Lee H, Jang Y, Lee YM, Lee H, Kim GH, Kim JJ (2015). Enhanced removal of PAHs by *Peniophora incarnata* and ascertainment of its novel ligninolytic enzyme genes. J Environ Manage.

[20] Lee AH, Kang CM, Lee YM, Lee H, Yun CW, Kim GH (2016). Heterologous expression of a new manganese-dependent peroxidase gene from *Peniophora incarnata* KUC8836 and its ability to remove anthracene in *Saccharomyces cerevisiae*. J Biosci Bioeng.

[21] Lee H, Yun SY, Jang S, Kim G-H, Kim J-J (2015). Bioremediation of polycyclic aromatic hydrocarbons in creosote-contaminated soil by *Peniophora incarnata* KUC8836. Bioremediat J.

[22] Lee H, Jang Y, Choi YS, Kim MJ, Lee J, Lee H (2014). Biotechnological procedures to select white rot fungi for the degradation of PAHs. J Microbiol Methods.

[23] Zhang L, You T, Zhou T, Zhang L, Xu F (2016). Synergistic effect of white-rot fungi and alkaline pretreatments for improving enzymatic hydrolysis of poplar wood. Ind Crops Prod.

[24] Lee JW, Gwak KS, Park JY, Park MJ, Choi DH, Kwon M (2007). Biological pretreatment of softwood *Pinus densiflora* by three white rot fungi. J Microbiol.

[25] Yu H, Guo G, Zhang X, Yan K, Xu C (2009). The effect of biological pretreatment with the selective white-rot fungus *Echinodontium taxodii* on enzymatic hydrolysis of softwoods and hardwoods. Bioresour Technol.

[26] Agbor VB, Cicek N, Sparling R, Berlin A, Levin DB (2011). Biomass pretreatment: fundamentals toward application. Biotechnol Adv.

[27] Castoldi R, Bracht A, de Morais GR, Baesso ML, Correa RCG, Peralta RA (2014). Biological pretreatment of *Eucalyptus grandis* sawdust with white-rot fungi: Study of degradation patterns and saccharification kinetics. Chem Eng J.

[28] Gabhane J, William SP, Vaidya AN, Das S, Wate SR (2015). Solar assisted alkali pretreatment of garden biomass: Effects on lignocellulose degradation, enzymatic hydrolysis, crystallinity and ultra-structural changes in lignocellulose. Waste Manag.

[29] Xu C, Ma F, Zhang X, Chen S (2010). Biological pretreatment of corn stover by *Irpex lacteus* for enzymatic hydrolysis. J Agric Food Chem.

[30] Huberman LB, Liu J, Qin L, Glass NL (2016). Regulation of the lignocellulolytic response in filamentous fungi. Fungal Biol Rev.

[31] Dong M, Wang S, Xu F, Wang J, Yang N, Li Q (2019). Pretreatment of sweet sorghum straw and its enzymatic digestion: insight into the structural changes and visualization of hydrolysis process. Biotechnol Biofuels.

[32] Wang R, You T, Yang G, Xu F (2017). Efficient short time white rot-brown rot fungal pretreatments for the enhancement of enzymatic saccharification of corn cobs. ACS Sustainable Chem Eng.

[33] Shirkavand E, Baroutian S, Gapes DJ, Young BR (2017). Pretreatment of radiata pine using two white rot fungal strains *Stereum hirsutum* and *Trametes versicolor*. Energy Convers Manage.

[34] Kong W, Fu X, Wang L, Alhujaily A, Zhang J (2017). A novel and efficient fungal delignification strategy based on versatile peroxidase for lignocellulose bioconversion. Biotechnol Biofuels.

[35] Yan K, Liu F, Chen Q, Ke M, Huang X, Hu W (2016). Pyrolysis characteristics and kinetics of lignin derived from enzymatic hydrolysis residue of bamboo pretreated with white-rot fungus. Biotechnol Biofuels.

[36] Del Rıo JC, Gutiérrez A, Martınez MJ, Martınez AT (2001). Py–GC/MS study of *Eucalyptus globulus* wood treated with different fungi. J Anal Appl Pyrol.

[37] Singh D, Zeng J, Laskar DD, Deobald L, Hiscox WC, Chen S (2011). Investigation of wheat straw biodegradation by *Phanerochaete chrysosporium*. Biomass Bioenergy.

[38] Skyba O, Douglas CJ, Mansfield SD (2013). Syringyl-rich lignin renders poplars more resistant to degradation by wood decay fungi. Appl Environ Microbiol.

[39] Vasco-Correa J, Luo X, Li Y, Shah A (2019). Comparative study of changes in composition and structure during sequential fungal pretreatment of non-sterile lignocellulosic feedstocks. Ind Crops Prod.

[40] Martínez AT, Camarero S, Gutiérrez A, Bocchini P, Galletti GC. Studies on wheat lignin degradation by *Pleurotus* species using analytical pyrolysis. J Anal Appl Pyrol. 2001. 58:401–411.

[41] Khan MU, Ahring BK (2019). Lignin degradation under anaerobic digestion: Influence of lignin modifications -A review. Biomass Bioenergy.

[42] Sutzl L, Foley G, Gillam EMJ, Boden M, Haltrich D (2019). The GMC superfamily of oxidoreductases revisited: analysis and evolution of fungal GMC oxidoreductases. Biotechnol Biofuels.

[43] Dashtban M, Schraft H, Syed TA, Qin W (2010). Fungal biodegradation and enzymatic modification of lignin. Int J Biochem Mol Biol.

[44] Bodeker IT, Nygren CM, Taylor AF, Olson A, Lindahl BD (2009). ClassII peroxidase-encoding genes are present in a phylogenetically wide range of ectomycorrhizal fungi. ISME J.

[45] Ma J, Zhang K, Liao H, Hector SB, Shi X, Li J (2016). Genomic and secretomic insight into lignocellulolytic system of an endophytic bacterium *Pantoea ananatis* Sd-1. Biotechnol Biofuels.

[46] Gao D, Uppugundla N, Chundawat SP, Yu X, Hermanson S, Gowda K (2011). Hemicellulases and auxiliary enzymes for improved conversion of lignocellulosic biomass to monosaccharides. Biotechnol Biofuels.

[47] Hemsworth GR, Johnston EM, Davies GJ, Walton PH (2015). Lytic Polysaccharide Monooxygenases in Biomass Conversion. Trends Biotechnol.

[48] Hou X, Liu Q, Meng Q, Wang L, Yan H, Zhang L (2020). TMT-based quantitative proteomic analysis of porcine muscle associated with postmortem meat quality. Food Chem.

[49] Perez-Riverol Y, Csordas A, Bai J, Bernal-Llinares M, Hewapathirana S, Kundu DJ (2019). The PRIDE database and related tools and resources in 2019: improving support for quantification data. Nucleic Acids Res.

[50] Mortazavi A, Williams BA, McCue K, Schaeffer L, Wold B (2008). Mapping and quantifying mammalian transcriptomes by RNA-Seq. Nat Methods.

[51] Love MI, Huber W, Anders S (2014). Moderated estimation of fold change and dispersion for RNA-seq data with DESeq2. Genome Biol.

[52] Sluiter A, Hames B, Ruiz R, Scarlata C, Sluiter J, Templeton D (2008). Determination of structural carbohydrates and lignin in biomass. Laboratory Analytical Procedure.

[53] Ma J, Zhang K, Huang M, Hector SB, Liu B, Tong C (2016). Involvement of Fenton chemistry in rice straw degradation by the lignocellulolytic bacterium *Pantoea ananatis* Sd-1. Biotechnol Biofuels.

[54] Park S, Baker JO, Himmel ME, Parilla PA, Johnson DK (2010). Cellulose crystallinity index measurement techniques and their impact on interpreting cellulase performance. Biotechnol Biofuels.

