# Supplementary figures and images for: Selective delignification of poplar wood with a newly isolated white-rot basidiomycete Peniophora incarnata T-7 by submerged fermentation to enhance saccharification
Source: Biotechnol Biofuels. 2021 Jun 12;14:135. doi: 10.1186/s13068-021-01986-y (PMC8199694; doi:10.1186/s13068-021-01986-y)

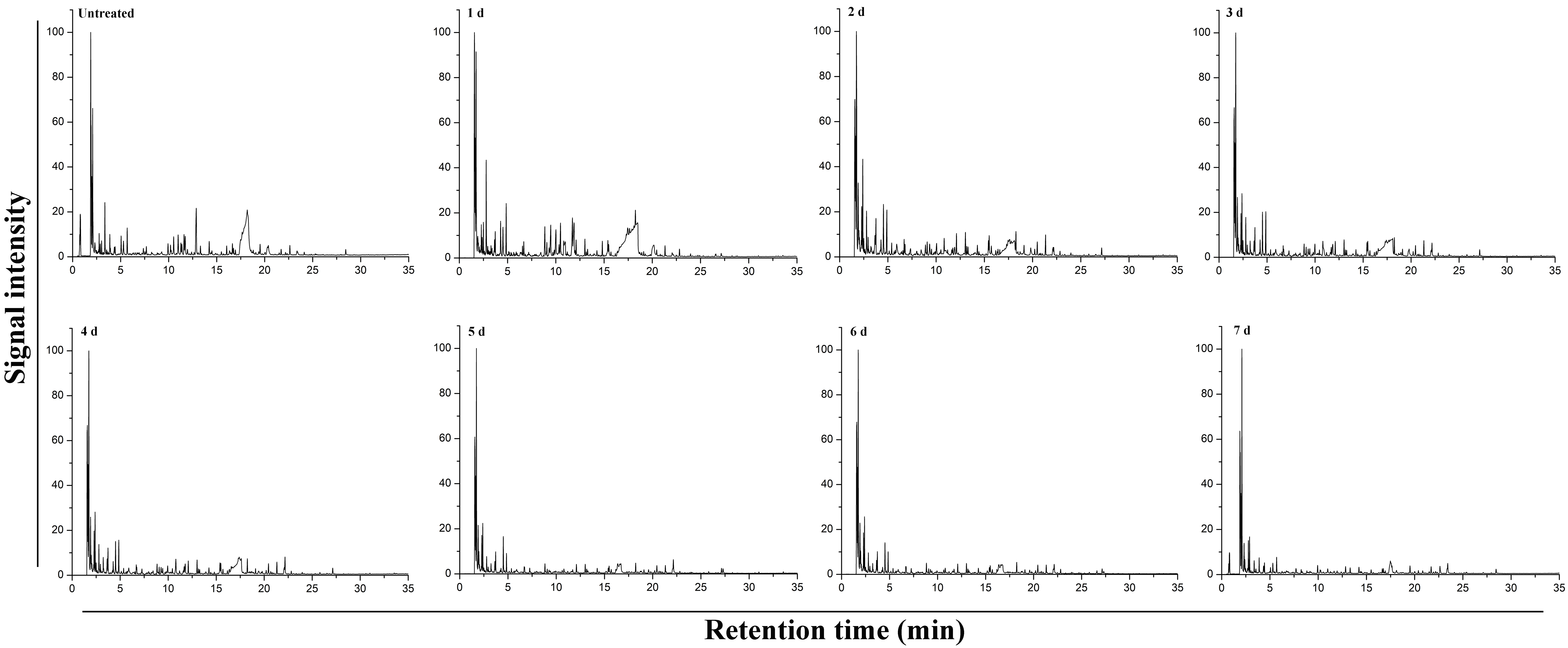

Supplement: Supplementary file 1 — Additional file 1: Figure S1. Qualitative detection of P. incarnate T-7 degradation ability for guaiacol and Azure B substrates. A, B, guaiacol-containing PDA medium; C and D, Azure B-containing PDA medium. [file 13068_2021_1986_MOESM1_ESM.png]

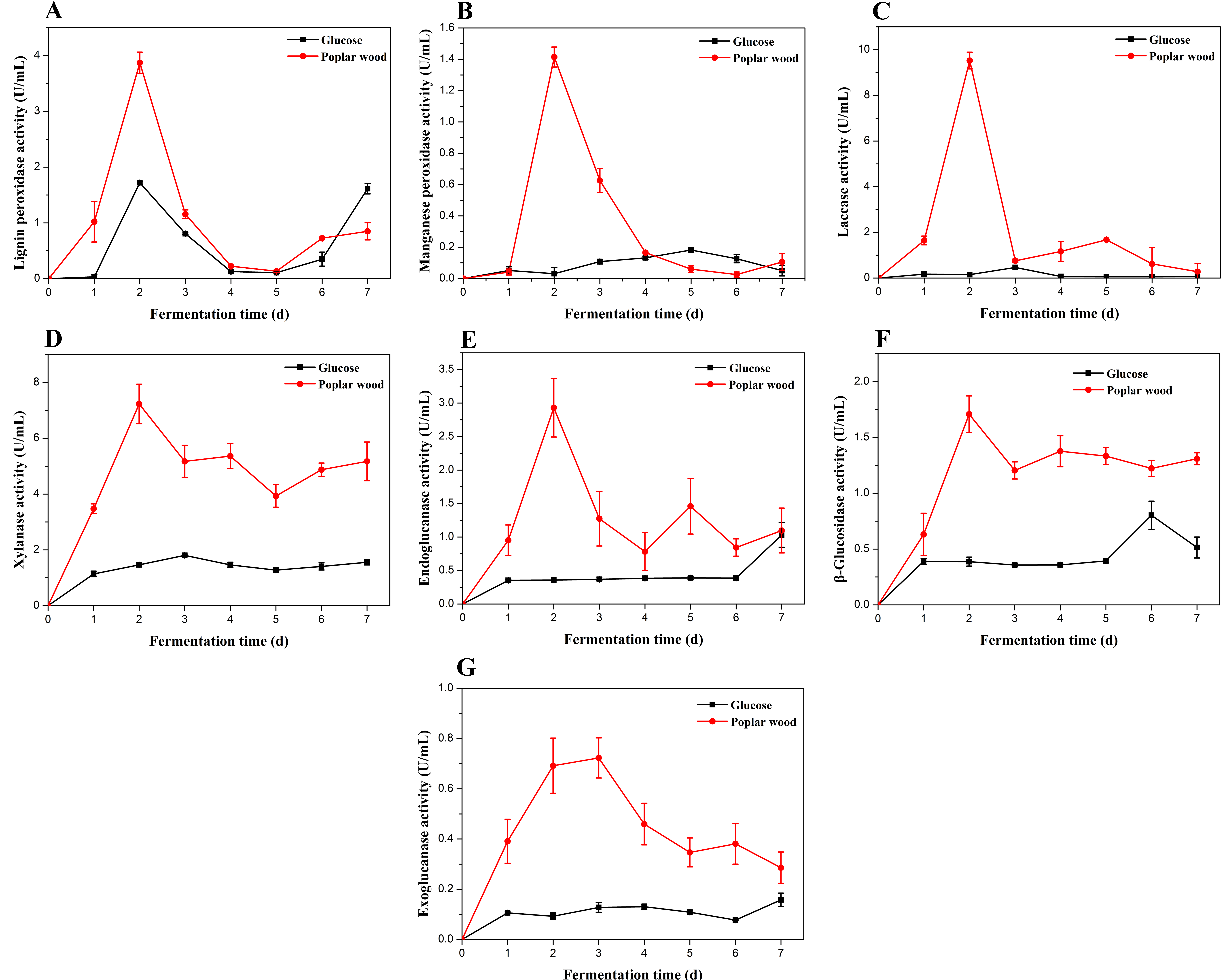

Supplement: Supplementary file 8 — Additional file 8: Classification of identified proteins from secretome of P. incarnate T-7 based on biological function. [file 13068_2021_1986_MOESM8_ESM.png]

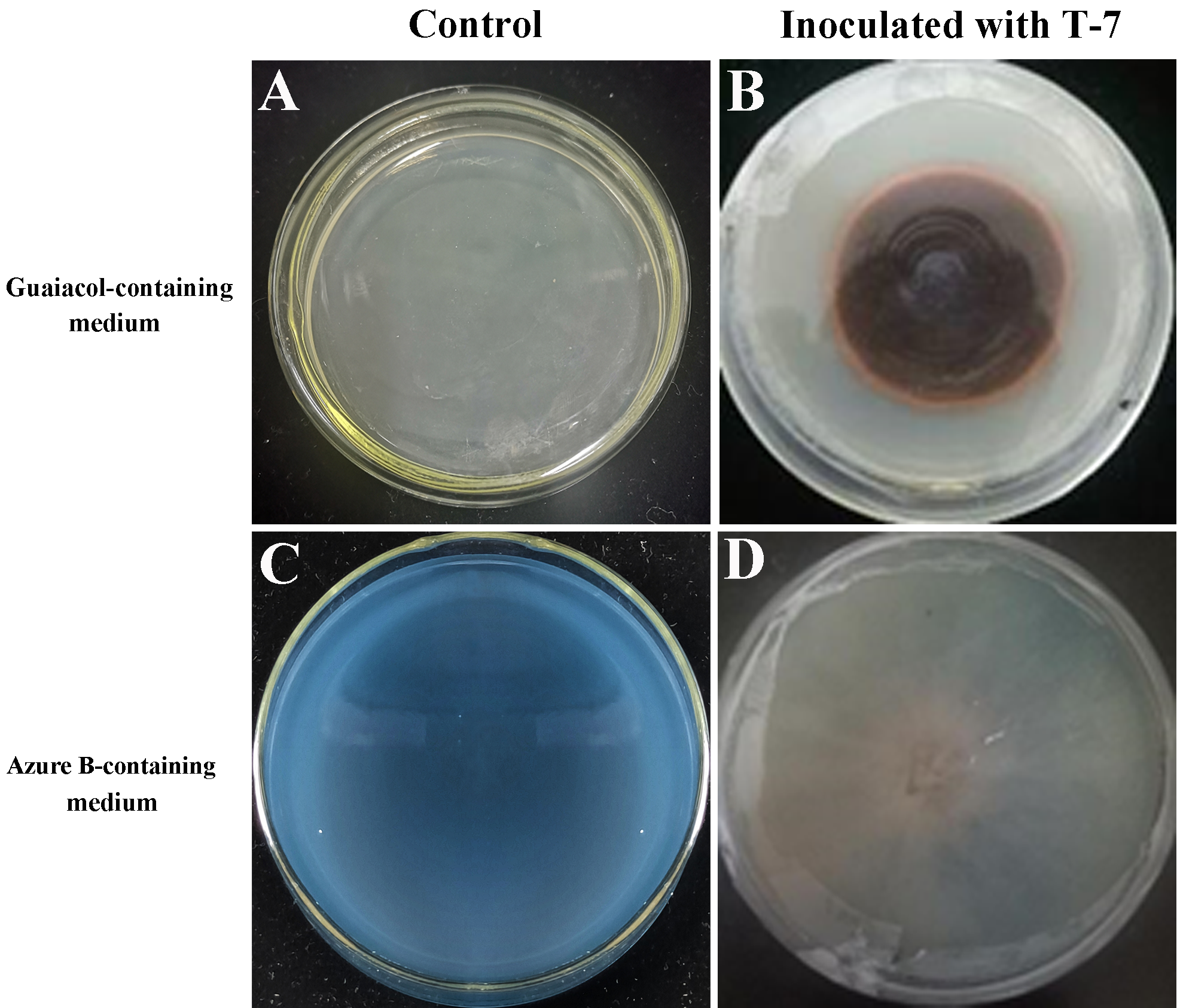

Supplement: Supplementary file 11 — Additional file 11: Table S2. Percentage numbers of the abundant annotated species from transcriptomic sequencing. [file 13068_2021_1986_MOESM11_ESM.png]
