# Supplementary material for: Selective delignification of poplar wood with a newly isolated white-rot basidiomycete Peniophora incarnata T-7 by submerged fermentation to enhance saccharification
Source: Biotechnol Biofuels. 2021 Jun 12;14:135. doi: 10.1186/s13068-021-01986-y (PMC8199694; doi:10.1186/s13068-021-01986-y)
Supplement: Supplementary file 2 — Additional file 2: Figure S2. The pyrograms of poplar wood samples before and after treated by P. incarnate T-7 with different time. [file 13068_2021_1986_MOESM2_ESM.docx]

**Table S2** The lignin-derived pyrolysates from poplar wood samples before and after treated by *P*. *incarnate* T-7 with different time identified by Py-GC/MS with relative peak areas.

| Retention time (min) | Compound | Group | 0 | 1 | 2 | 3 | 4 | 5 | 6 | 7 |
| --- | --- | --- | --- | --- | --- | --- | --- | --- | --- | --- |
| 5.2 | Benzenol | H | 0.99 | 0.56 | 0.35 | 0.28 | 0.21 | 0.34 | ND | ND |
| 6.8 | Methylphenol | H | 0.12 | ND | 0.24 | 0.16 | 0.09 | 0.23 | 0.07 | ND |
| 7.6 | Mequinol | G | 0.42 | 0.47 | ND | ND | 0.28 | 0.22 | 0.16 | 0.33 |
| 9.2 | Benzeneformic acid | H | ND | 0.23 | ND | 0.26 | 0.14 | 0.21 | 0.14 | 0.12 |
| 10.1 | Methylguaiacol | G | 0.42 | 0.37 | 0.05 | 0.32 | 0.47 | 0.37 | 0.25 | 0.25 |
| 10.2 | 1,2-Benzenediol | H | 0.23 | 0.28 | 0.14 | 0.17 | 0.15 | 0.27 | 0.18 | 0.24 |
| 11.8 | 1,2-Dihydroxy-3-methylbenzene | H | 0.04 | 0.12 | 0.03 | 0.11 | 0.17 | ND | 0.23 | 0.07 |
| 11.9 | 1,4-Benzenediol, 2-methoxy- | G | 0.31 | 0.36 | 0.27 | 0.35 | 0.24 | 0.17 | 0.09 | 0.10 |
| 12.3 | p-Ethylguaiacol | G | 0.13 | 0.11 | 0.36 | 0.27 | 0.38 | 0.25 | 0.14 | 0.12 |
| 12.6 | 3,4-Dihydroxytoluene | H | 0.15 | 0.13 | 0.08 | 0.19 | ND | 0.17 | 0.16 | 0.12 |
| 13.3 | 2-Methoxy-4-vinylphenol | G | 0.41 | 0.56 | 0.39 | 0.46 | 0.42 | 0.36 | 0.27 | 0.21 |
| 13.4 | 3-Methoxy-5-methylphenol | H | 0.06 | 0.12 | 0.06 | 0.12 | 0.19 | 0.13 | 0.08 | ND |
| 14.2 | 3,4-Dimethoxyphenol | S | 0.73 | 0.69 | 0.53 | 0.49 | 0.55 | 0.48 | 0.57 | 0.86 |
| 14.4 | 3-Allyl-2-methoxyphenol | G | 0.08 | 0.13 | 0.25 | 0.14 | 0.23 | ND | 0.12 | 0.11 |
| 14.5 | 3,4-Dimethoxyphenyl acetate | S | 0.25 | 0.28 | 0.94 | 0.65 | 0.48 | 0.36 | 0.25 | 0.31 |
| 15.4 | Vanillin | G | 0.21 | 0.15 | ND | 0.23 | 0.28 | 0.32 | 0.16 | 0.06 |
| 15.7 | Phenol, 2-methoxy-5-propenyl- | G | 0.06 | 0.26 | 0.11 | ND | ND | 0.13 | 0.09 | 0.10 |
| 16.4 | 1-Phenyl-2-pentanone | H | 0.15 | 0.07 | 0.13 | 0.08 | 0.12 | ND | ND | ND |
| 16.6 | 3,5-Dimethoxy-4-hydroxytoluene | S | 0.63 | 0.88 | 0.69 | 0.48 | ND | 0.37 | 0.48 | 0.62 |
| 16.7 | Isoeugenol | G | 0.35 | 0.31 | 0.38 | 0.41 | 0.35 | 0.29 | 0.16 | 0.2 |
| 16.8 | o-Methoxycinnamaldehyde | S | ND | 0.34 | 0.43 | 0.26 | ND | ND | ND | 0.18 |
| 16.9 | Propylguaiacol | G | 0.30 | 0.22 | 0.36 | 0.27 | 0.28 | 0.14 | 0.23 | 0.13 |
| 17.4 | p-Hydroxybenzalacetone | H | ND | 0.26 | 0.14 | 0.23 | 0.16 | 0.28 | 0.27 | 0.72 |
| 17.6 | Hydroxybenzalacetone | S | 6.64 | 5.73 | 4.94 | 2.55 | 3.61 | 3.75 | 4.58 | 0.99 |
| 17.8 | 3-Methoxy-4,5,6-trimethylphenol | S | 5.76 | 4.67 | 3.42 | 5.52 | 4.72 | 3.14 | ND | ND |
| 18.1 | p-Hydroxybenzoic acid | H | ND | 0.15 | 0.05 | 0.11 | 0.21 | 0.25 | 0.26 | 0.31 |
| 18.5 | Benzene, 1,2,3-trimethoxy-5-methyl- | S | 0.09 | 0.11 | ND | 0.07 | 0.13 | 0.07 | 0.16 | ND |
| 18.7 | Guaiacylacetone | G | 0.06 | 0.07 | 0.14 | 0.04 | 0.16 | ND | ND | ND |
| 19.5 | 3,5-Dimethoxyacetophenone | S | 0.64 | 0.97 | 1.26 | 1.47 | 0.89 | 0.67 | 0.74 | 1.08 |
| 20.0 | Benzene, 1,2-dimethoxy-4-(2-propenyl)- | G | ND | 0.12 | 0.18 | 0.24 | 0.15 | 0.26 | 0.23 | 0.24 |
| 20.3 | Phenol, 2,6-dimethoxy-4-(2-propenyl)-) | G | 0.93 | 0.84 | 0.49 | 0.36 | 0.58 | 0.44 | 0.13 | 0.12 |
| 21.5 | 1,2-Dimethoxy-4-(1-methoxyethenyl)benzene | G | 0.13 | 0.15 | 0.23 | 0.29 | 0.17 | 0.11 | 0.34 | 0.23 |
| 21.7 | 3,4-Dimethoxy-5-hydroxybenzaldehyde | S | 0.34 | 0.63 | ND | 0.34 | 0.46 | 0.32 | 0.58 | 0.89 |
| 21.9 | 4-((1E)-3-Hydroxy-1-propenyl)-2-methoxyphenol | G | ND | ND | 0.11 | 0.07 | 0.16 | 0.14 | 0.28 | 0.19 |
| 22.1 | Coniferaldehyde | G | 0.24 | ND | 0.37 | 0.44 | 0.34 | 0.25 | ND | 0.22 |
| 22.6 | Methoxyeugenol | G | 0.46 | 0.36 | 0.26 | ND | 0.17 | 0.24 | 0.33 | 0.41 |
| 23.3 | 3,4,5-Trimethoxybenzaldehyde | S | ND | ND | ND | 0.17 | 0.26 | 0.13 | 0.24 | 0.78 |
| 23.3 | 4-Hydroxy-2-methoxycinnamaldehyde | S | 0.34 | 0.25 | 0.47 | ND | ND | 0.24 | 0.17 | ND |
| 23.4 | 4-(1-Hydroxyallyl)-2-methoxyphenol | G | 0.22 | 0.17 | 0.43 | 0.26 | ND | 0.15 | 0.25 | 0.33 |
| 24.1 | 3,5-Dimethoxy-4-hydroxyphenylacetic acid | S | 0.16 | 0.26 | 0.38 | 0.27 | 0.18 | 0.14 | 0.23 | 0.21 |
| 25.3 | 3,5-Dimethoxy-4-hydroxycinnamaldehyde | S | ND | 0.24 | 0.33 | 0.41 | 0.35 | 0.27 | ND | 0.22 |
| 27.9 | 1,2-Benzenedicarboxylic acid | H | ND | ND | 0.09 | 0.12 | 0.08 | ND | 0.12 | 0.06 |
| 28.4 | 2,5-Dimethoxycinnamic acid | S | 0.34 | 0.26 | 1.18 | 0.96 | 0.64 | 0.58 | 0.61 | 0.74 |
|  | Total peaks areas of lignin derivates |  | 22.39 | 21.88 | 20.26 | 19.62 | 18.45 | 16.24 | 13.35 | 11.87 |
|  | Total peak areas of H group |  | 1.74 | 1.92 | 1.31 | 1.83 | 1.52 | 1.88 | 1.51 | 1.64 |
|  | Total peak areas of S group |  | 15.92 | 15.31 | 14.57 | 13.64 | 12.27 | 10.52 | 8.61 | 6.88 |
|  | Total peak areas of G group |  | 4.73 | 4.65 | 4.38 | 4.15 | 4.66 | 3.84 | 3.23 | 3.35 |
|  | S/G |  | 3.37 | 3.29 | 3.33 | 3.29 | 2.63 | 2.74 | 2.67 | 2.05 |
