# Supplementary material for: Selective delignification of poplar wood with a newly isolated white-rot basidiomycete Peniophora incarnata T-7 by submerged fermentation to enhance saccharification
Source: Biotechnol Biofuels. 2021 Jun 12;14:135. doi: 10.1186/s13068-021-01986-y (PMC8199694; doi:10.1186/s13068-021-01986-y)
Supplement: Supplementary file 10 — Additional file 10: Figure S4. Hierarchical clustering of expression of lignocellulolytic enzymes including lignin-degrading enzymes (A), hemicellulases (B) and cellulases (C) encoding transcripts by P. incarnate T-7 on poplar wood substrate and glucose substrate, respectively. [file 13068_2021_1986_MOESM10_ESM.docx]

**Table S2** Percentage numbers of the abundant annotated species

| Species | Gene number | Percentage |
| --- | --- | --- |
| *Peniophora* sp. | 9987 | 93.64% |
| *Heterobasidion irregulare* | 79 | 0.74% |
| *Dentipellis fragilis* | 69 | 0.65% |
| *Stereum hirsutum* | 44 | 0.41% |
| *Hericium alpestre* | 35 | 0.33% |
| *Plicaturopsis crispa* | 20 | 0.19% |
| *Sphaerobolus stellatus* | 18 | 0.17% |
| *Mucor ambiguus* | 14 | 0.13% |
| *Heliocybe sulcata* | 11 | 0.10% |
| other | 388 | 36.34% |
